# Biophysical Approaches Facilitate Computational Drug Discovery for ATP-Binding Cassette Proteins

**DOI:** 10.1155/2017/1529402

**Published:** 2017-03-19

**Authors:** Steven V. Molinski, Zoltán Bozóky, Surtaj H. Iram, Saumel Ahmadi

**Affiliations:** ^1^Faculty of Medicine, University of Toronto, Toronto, ON, Canada; ^2^Department of Chemistry & Biochemistry, College of Arts and Sciences, South Dakota State University, Brookings, SD, USA

## Abstract

Although membrane proteins represent most therapeutically relevant drug targets, the availability of atomic resolution structures for this class of proteins has been limited. Structural characterization has been hampered by the biophysical nature of these polytopic transporters, receptors, and channels, and recent innovations to in vitro techniques aim to mitigate these challenges. One such class of membrane proteins, the ATP-binding cassette (ABC) superfamily, are broadly expressed throughout the human body, required for normal physiology and disease-causing when mutated, yet lacks sufficient structural representation in the Protein Data Bank. However, recent improvements to biophysical techniques (e.g., cryo-electron microscopy) have allowed for previously “hard-to-study” ABC proteins to be characterized at high resolution, providing insight into molecular mechanisms-of-action as well as revealing novel druggable sites for therapy design. These new advances provide ample opportunity for computational methods (e.g., virtual screening, molecular dynamics simulations, and structure-based drug design) to catalyze the discovery of novel small molecule therapeutics that can be easily translated from computer to bench and subsequently to the patient's bedside. In this review, we explore the utility of recent advances in biophysical methods coupled with well-established in silico techniques towards drug development for diseases caused by dysfunctional ABC proteins.

## 1. Introduction

The adenosine triphosphate- (ATP-) binding cassette (ABC) protein superfamily is comprised of transmembrane proteins that utilize the energy generated from ATP binding and hydrolysis to translocate physiological solutes or ions across the lipid bilayers of certain cell types throughout the body [1]. Based on the directionality of transport, ABC transporters can be classified as importers (bringing solutes into the cell) or exporters (expelling solutes from the cell). Both importer and exporter ABC proteins are present in bacteria and archaea, whereas only exporters are found in eukaryotes [2–4]. In humans, the importance of ABC transporters is highlighted by the fact that mutations in many members of the superfamily have been associated with diseases [1, 5]. Importantly, many ABC proteins are also involved in the absorption, distribution, and excretion of xenobiotics, as they mediate efflux of these biomolecules and their metabolites across certain tissues [1, 6]. Further, several ABC proteins have demonstrated importance in mediating multidrug resistance, such that overexpression of these members in cancerous tissues prevents accumulation of chemicals (i.e., chemotherapeutics) via active transport, leading to subsequent relapse and cancer progression [1, 7]. Taken together, it is clear that ABC proteins have an important role in various physiological processes and associated human diseases, whereas, in bacteria and archaea, ABC importers/exporters are essential for uptaking nutrients or effluxing toxic molecules, respectively, across cell membranes.

**Figure 1:**
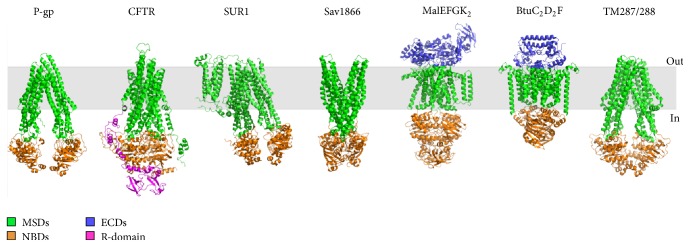
*Architecture of prototypic human and bacterial ABC proteins*. Human (P-gp/ABCB1, CFTR/ABCC7), rat (SUR1/ABCC8), and bacterial (Sav1866, MalEFGK_2_, BtuC_2_D_2_F, and TM287/288) ABC proteins are shown relative to the plasma membrane [8–13]. P-gp is a multidrug transporter, CFTR is a unique chloride channel, SUR1 is a regulator of an inwardly rectifying potassium channel, Sav1866 is an exporter, MalEFGK_2_ is a type I importer, BtuC_2_D_2_F is a type II importer, and TM287/288 is an exporter. MSDs are shown in green, NBDs are in orange, extracellular domains (ECMs) are in blue, R-domain is in pink, and the phospholipid bilayer is in gray. The core structure of an ABC protein consists of MSD1, NBD1, MSD2, and NBD2; however, SUR1/ABCC8 contains an additional MSD0. The full-length homology model of human CFTR is shown [14].

The human ABC protein superfamily is comprised of 49 genes [5]. Most of these are efflux transporters; however certain members of the ABC protein superfamily are unique. For instance, the Cystic Fibrosis transmembrane conductance regulator (CFTR/ABCC7) is a phospho-regulated chloride channel, while the sulfonylurea receptors 1 and 2 (SUR1/ABCC8 and SUR2/ABCC9, resp.) are regulators of inwardly rectifying potassium channels [15, 16]. Nonetheless, the core structural architecture of ABC proteins is comprised of two membrane-spanning domains (MSDs) and two nucleotide-binding domains (NBDs), although certain “outlier” members contain an additional MSD (e.g., MSD0 of MRP1/ABCC1 and SUR1/ABCC8) or regulatory domain (i.e., R-domain of CFTR/ABCC7) (Figure 1) [17–20]. Based on the available structural data, three types of ABC transporter families have been characterized: (1) type I importers, (2) type II importers, and (3) exporters. In the case of type I and II ABC importers, certain members require a specific solute-binding protein present in the periplasm, which is responsible for substrate specificity and further coordinates the delivery of substrates to the transporter [2, 4, 21]. Among these importers, the best characterized ABC transporter system is the maltose transporter, where several structural intermediates have been captured by X-ray crystallography [8, 22, 23]. In comparison to importers, the ABC exporters have long transmembrane helices that extend into the cytoplasm as well as the so-called “coupling-helix” that provides an interface with the cytoplasmic NBDs [24]. This interface region seems to be a hot-spot for structural stability, proper folding, and assembly of different ABC transporters including CFTR (ABCC7) and multidrug resistance proteins (MRPs) and furthermore a location enriched in disease-causing mutations [6, 18, 25, 26].

ABC proteins responsible for multidrug resistance in human cancers include the multidrug resistance protein 1 (MRP1/ABCC1), P-glycoprotein (P-gp/ABCB1), and the breast cancer resistance protein (BCRP/ABCG2) [7, 27–29]. In specific tumour subtypes, these proteins are overexpressed and prevent sufficient “cell killing” (i.e., apoptosis/necrosis) via chemotherapeutics. Thus, small molecule therapies designed to inhibit these efflux pumps are of great interest to the medical community as potential adjuvants to chemotherapy. Accordingly, atomic resolution structures of these three ABC proteins would be advantageous for medicinal chemists as well as computational biologists, further guiding rational in silico and subsequently in vitro, quantitative structure-activity relationship (QSAR) studies and structure-based drug design (SBDD) initiatives [30, 31].

Design of small molecule modulators for other disease-relevant ABC protein targets (e.g., CFTR/ABCC7, TAP1/ABCB2, and TAP2/ABCB3; Table 1) could also be facilitated once biophysical methodologies enable more efficient characterization of these polytopic and highly dynamic membrane proteins. To date, approximately 670 membrane protein structures have been deposited in the Protein Data Bank [32]. However, most of these are from non-human species, including other mammals (mainly rodents), archaea, bacteria, fungi, parasites, and plants. Thus, in silico generation of homology models is typically necessary to capture the structural specificities of human membrane proteins, and this is not different for the ABC superfamily. In addition, it is important to note that of these ~670 membrane protein structures (among a database of approximately 125,000 structures) only 37 of these are ABC proteins (~0.5% of membrane proteins or ~0.03% of all structurally characterized proteins), and most have been deposited in recent years; further, only five full-length human ABC proteins have been structurally characterized to date [32, 33]. Therefore, although these proteins are therapeutically relevant, they are indeed structurally underrepresented. By overcoming challenges associated with the complexities of their structural arrangement and dynamics in biological systems, high resolution structures of ABC proteins associated with human diseases will become more readily available. This would provide sufficient insight into their structure-function relationships, as well as identify novel druggable pockets to aid in the in silico design of pharmacological chaperones, and/or initiation of virtual screening campaigns, in order to repair mutant ABC proteins towards the wild-type conformation.

To date, certain biophysical methods have been preferentially used to characterize the currently available ABC proteins, and these include X-ray crystallography, cryo-electron microscopy (cryo-EM), and nuclear magnetic resonance (NMR) spectroscopy. Each technique has its own set of advantages and disadvantages, and these will be explored in more detail in Table 2. In brief, X-ray crystallography has been the primary technique of choice, as it was used to resolve the first protein structure (i.e., myoglobin in 1960; [34]) and has subsequently been extensively used for soluble proteins, as well as some membrane proteins. On the other hand, NMR spectroscopy, in practice since the early 1980s, has been mainly utilized to understand protein dynamics and folding, including that of certain membrane proteins [35]. However, the relatively “new” (late 1980s) method, cryo-EM, is becoming more popular and powerful, especially due to the recent improvements in electron detectors and accompanying three-dimensional protein reconstruction software [36–38]. Importantly, unlike X-ray crystallography, cryo-EM does not require proteins to form crystal lattices and thus can be used to investigate asymmetric nature and distinct conformational spectrum of proteins such as those in the ABC superfamily [19, 39]. Accordingly, additional innovations to the cryo-EM workflow have allowed for higher resolution structures of increasingly complex protein samples (e.g., bearing posttranslational modifications, ligands, and macromolecular binding partners) to be achieved, including those of several disease-relevant and plasma membrane-associated ABC proteins in recent years [40, 41]. Appropriately, today's structural biology pursuits have been aptly named a “cryo-EM revolution” for drug discovery [36–38].

## 2. In Silico Methods Facilitate Drug Discovery for Disease-Associated ABC Proteins

**Figure 2:**
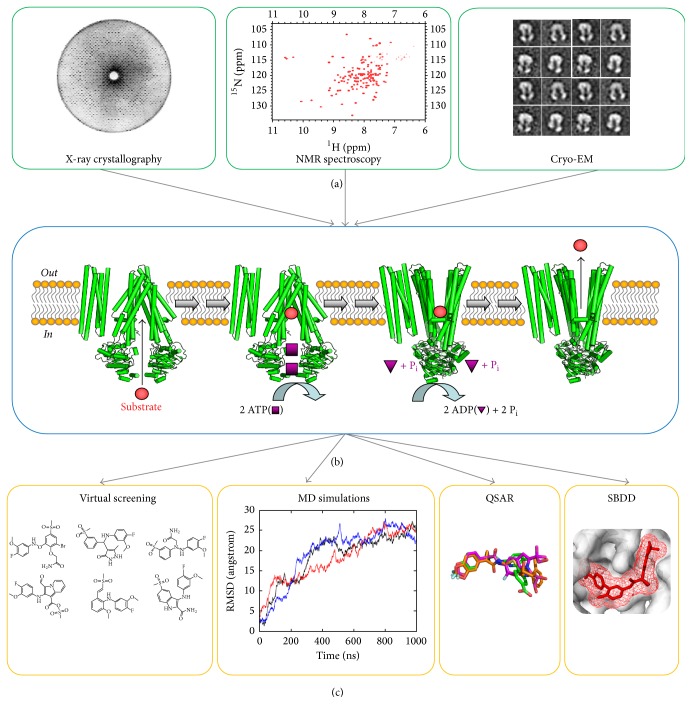
*Experimental workflow from biophysical characterization of ABC proteins in multiple conformational states to in silico discovery and design of small molecule modulators*. Biophysical techniques are shown in green boxes (a), multiple conformations of a representative ABC protein are shown in the blue box (b), and in silico methods are shown in orange boxes (c). The cryo-EM image is modified from [39].

Using solved structures of disease-associated ABC proteins, many of the traditional in silico structure-based tools can be used to facilitate drug development (Figure 2). In general, for diseases caused by missense mutations to certain members of the ABC superfamily, the first step towards drug discovery and rational drug design is to model these side-chain variants onto the tertiary protein structure and subsequently conduct molecular dynamics (MD) simulations to “relax” the biomolecular system so that it reaches its lowest energy conformation within the biological constraints of a phospholipid bilayer environment [14, 52, 53]. With these energy-minimized mutant ABC protein structures, disease-relevant conformations are sampled, potentially enabling the identification of aberrant structural motifs as well as druggable pockets and further allowing for therapeutically relevant target-specificity with respect to the development of small molecule modulators.

However, as previously mentioned, most ABC proteins that have been structurally characterized and reported in the Protein Data Bank to date are from non-human species [32]. Thus, homology modeling is typically required at the onset of ABC-centric drug development initiatives. Many of the human models generated and published during the previous decade were based on X-ray crystal structures of homologous bacterial templates (e.g., Sav1866 and MsbA; [11, 54]), and these models have provided sufficient utility for drug development via in silico medicinal chemistry and virtual screening approaches, especially for Cystic Fibrosis (i.e., using CFTR/ABCC7 models; [55–58]). However, even though compounds identified in these in silico studies have had positive effects in vitro, to date, none have been translated to the clinic; therefore, caution must be taken when using modeled human structures to guide drug discovery efforts. Accordingly, the direct structural characterization of human ABC proteins is desired, as it would mitigate risks associated with using bacterial structure-based homology models (i.e., low sequence identity, structural diversity). Importantly, in certain aspects, cryo-EM has superseded the usefulness of X-ray crystallography and NMR spectroscopy in the ABC protein field over the past decade, since it has been recently, successfully, and frequently employed to resolve the complex, polytopic, and usually asymmetric nature of this protein superfamily (even human structures) at higher resolutions (and near full-length polypeptides) than previously possible [40, 41].

By using these high-quality, human disease-relevant biophysical structures, best-in-class therapies can be developed. Appropriate medicinal chemistry approaches facilitated by well-established in silico techniques, including homology modeling [59], MD simulations [60, 61], virtual screening [62, 63], QSAR [64, 65], and SBDD [66], can and have been used to drive therapeutic discovery for ABC proteins, mainly those requiring functional inactivation (i.e., ABCC1, ABCB1, and ABCG2 as previously mentioned). For example, inhibitors of ABCB1 have been identified using a combination of in silico techniques, and these small molecules are currently being evaluated in vitro as well as in vivo for inhibition of tumorigenesis [67, 68].

Another computational tool, which has provided much insight into the conformational dynamics, ATP-dependent transport/gating cycles, and putative mechanism-of-action of small molecule modulators of certain ABC proteins, is that of MD simulations [61]. This methodology considers both the biophysics and biochemistry of ABC proteins embedded in its native phospholipid bilayer environment, providing physiologically-relevant information about conformational dynamics as well as potential druggability. Thus, MD simulations can be used to sample conformational states which may be difficult to be structurally characterized using current biophysical methods, providing actionable information into the putative druggability of specific functional transition states. In addition, virtual screening is a complementary in silico tool which has been successfully used to identify and evaluate lead, drug-like small molecule candidates for several ABC proteins, mainly ABCB1 [62, 63]. Accordingly, chemical libraries of thousands to millions of compounds can be efficiently and computationally screened against an ABC protein target of interest with minimal upfront requirements of cost and time. This approach is typically used to narrow the chemical space of possible modulators to those with promising scaffolds. Furthermore, using this approach, lead candidates are ranked in terms of scaffold suitability and complementarity to the target of interest, and subsequently, representative chemicals are chosen for in vitro validation studies. Additional approaches which have demonstrated usefulness in solving the drug design problem involve QSAR and SBDD. QSAR aims to identify bioactive modulators by evaluating the predicted functional activity of a spectrum of drug derivatives [64, 65], while SBDD uses scaffolds of known modulators to precisely reposition/modify chemical moieties in order to improve predicted binding affinity and thus putative drug efficacy [66]. Importantly, these in silico approaches have been frequently used to the develop small molecule modulators of ABC transporters, mainly ABCB1, with several examples of success [64–66].

Lastly, any small molecules identified using these aforementioned in silico approaches must abide by previously established (and predictive) drug development algorithms, including Lipinski's “Rule of 5” [69] and the “Golden Triangle” [70]. Both parameters focus on the physicochemical properties of small molecules, mainly molecular weight, hydrophobicity (i.e., partition and distribution coefficients, logP, and logD, resp.), and calculated hydrogen-bond acceptors and donors. Lipinski's rules evaluate drug plausibility in terms of oral bioavailability in humans and consider a drug's pharmacokinetics (i.e., absorption, distribution, metabolism, and excretion), while the Golden Triangle aims to computationally optimize oral absorption/permeability as well as drug clearance. These algorithms classify compounds based on molecular weight and logD, in order to evaluate drug-likeness, efficacy, potency, clearance, and permeability, and any in silico-based discovery of a putative ABC protein modulator must meet these criteria in order to have potential therapeutic value in the clinic [71].

## 3. Therapy Development for Multidrug Resistance in Cancer, Cystic Fibrosis, and Herpes Simplex Virus Infection

As previously described, ABC proteins are found in all three domains of life (bacteria, archaea, and eukarya), playing many key physiological roles required for normal biology. ABCB1 is no exception; however, when this ABC transporter is overexpressed it is disease-causing in humans. ABCB1 was the first ABC protein to be identified and cloned, as it was highly expressed in the tumours of cancer patients [72]. ABCB1 is a polyspecific drug efflux pump, required for transport of physiological substrates and xenobiotics across certain tissues of the body, yet in cancer, this protein employs a survival response and becomes overexpressed in order to efflux chemotherapeutics [1, 7]. Thus, ABCB1 is an important target for adjuvant cancer therapy, such that specific inhibitors could potentially increase intracellular concentrations of cotreated chemotherapies; third generation ABCB1 inhibitors, developed in part using the biophysical structure of ABCB1 as a template for small molecule discovery, have shown efficacy towards tumorigenesis [7, 10].

Another ABC protein, CFTR (ABCC7), is the most unique member of the ABC superfamily. Cloning of the* CFTR* gene in conjunction with the discovery of CFTR as a phospho-regulated chloride channel was seminal in the development of high-throughput, target-based initiatives for Cystic Fibrosis drug discovery [15, 73–75]. Remarkably, these efforts lead to the development of two mutation-specific small molecule therapies for Cystic Fibrosis, a prototypic feat that would be much welcomed for other disease-associated ABC proteins [76]. Although the structure-based homology models of CFTR were important in classifying various experimental drugs based on their site of action on the tertiary structure of CFTR, a molecular understanding of drug synergy (achieved by one of the FDA-approved therapies: Orkambi®) could not be achieved using in silico tools alone [76, 77]. Furthermore, with the advent of recent innovations and improvements to cryo-EM-based techniques, the structure of CFTR was recently solved, paving the way for subsequent structure-based drug development of the next generation of more effective Cystic Fibrosis therapies [40].

In addition, the transporter associated with antigen processing (TAP1/ABCB2) is an important ABC family member required for adaptive immunity [78]. TAP1 transports foreign peptide antigens from invading pathogens into the endoplasmic reticulum of immune cells and allows these peptides to become displayed by the major histocompatibility complex on the cell surface following anterograde trafficking [78]. Transport to the plasma membrane allows recognition of foreign antigen by CD8+ T-cells via a macromolecular complex. This is relevant, as the herpes simplex virus is a unique pathogen which evades this immune response by expressing a peptide-based inhibitor of TAP1. Again, with the advent of recent innovations and improvements to cryo-EM-based techniques, the binding site of this inhibitor peptide was recently discovered and mechanism-of-action elucidated, further allowing for possible SBDD of small molecule modulators of the herpes simplex virus [40].

Finally, as more ABC proteins become structurally characterized in multiple conformations using state-of-the-art biophysical techniques, our understanding of similarities (and differences) between each unique family member will be further elucidated. This may facilitate identification of druggable binding sites which are only present in the mutant form of each target of interest, thereby allowing for efficient in silico virtual screening and SBDD initiatives aimed at developing ABC- and mutation-specific therapeutics. This approach may also assist with the repurposing of drugs within the ABC superfamily. One recent example is the repurposing of the CFTR/ABCC7-specific potentiator, ivacaftor (Kalydeco®), for patients with mutations in a homologous ABC protein: ABCB4 [79]. Both in silico and in vitro methods were synergistically used to validate this hypothesis, and furthermore, this finding highlights the importance of accurate structure-based classification of proteins and homologous family members with respect to translational drug discovery.

## 4. Conclusion

ABC proteins are required for normal physiology and can cause disease when mutated. Biophysical techniques aim to structurally characterize this family of membrane proteins, and recent improvements to certain techniques, mainly cryo-EM, have elucidated the tertiary topology of several disease-relevant ABC proteins. This structural information provides a foundation for in silico-based drug discovery and drug development initiatives (e.g., virtual screening and QSAR) intended to repair misfolded and/or dysfunctional mutant ABC variants. Thus, potentially druggable sites can be identified and investigated with respect to the compatibility of small molecule therapeutics aimed at improving structural defects caused by various mutations, and further, specific modulators can be designed using various well-established in silico approaches. Taken together, the advent of high resolution structures of these human disease-relevant proteins provided ample opportunity to translate findings from the lab into the clinic.

## Figures and Tables

**Table 1:** Relative characterization of ABC proteins that cause human diseases when mutated.

Gene	Protein	Endogenous substrate(s)	Disease	Publications on disease	Structure(s) in Protein Data Bank	Ref.
*ABCA1*	—	Cholesterol, phospholipids	Tangier disease	>700	N	[1]
*ABCA3*	—	Lipids, cholesterol	Newborn respiratory distress syndrome	>16,000	N	[42]
*ABCA4*	—	Vitamin A derivatives	Stargardt disease	>500	N	[1]
*ABCA12*	—	Lipids	Harlequin-type ichthyosis	>1,000	N	[43]
*ABCB2*	TAP1	Cytosolic peptides	Ankylosing spondylitis	>16,000	Y (cryo-EM, X-ray)	[40, 44, 45]
*ABCB3*	TAP2	Cytosolic peptides	Ankylosing spondylitis	>16,000	Y (cryo-EM)	[40, 45]
*ABCB4*	MDR2	Phospholipids	Progressive familial intrahepatic cholestasis type 3	>100	N	[1]
*ABCB7*	—	Heme, iron-sulfur clusters	X-linked sideroblastosis and anemia	>30	N	[1]
*ABCB11*	BSEP	Taurocholate, cholate conjugates	Progressive familial intrahepatic cholestasis type 2	>100	N	[1]
*ABCC2*	MRP2	Organic anions	Dubin-Johnson syndrome	>800	N	[46]
*ABCC6*	MRP6	Organic anions	Pseudoxanthoma elasticum	>1,700	N	[47]
*ABCC7*	CFTR	Chloride, bicarbonate	Cystic Fibrosis	>45,000	Y (cryo-EM, X-ray, NMR)	[15, 41, 48, 49]
*ABCC8*	SUR1	Sulfonylurea	Familial persistent hyperinsulinemic hypoglycemia of infancy	>100	Y (cryo-EM)	[1, 9]
*ABCC9*	SUR2	Sulfonylurea	Dilated cardiomyopathy with ventricular tachycardia	>100	N	[1]
*ABCD1*	ALD	Fatty acids	Adrenoleukodystrophy	>2,000	N	[1]
*ABCG5*	—	Sterols	Sitosterolemia	>200	Y (X-ray)	[50, 51]
*ABCG8*	—	Sterols	Sitosterolemia	>200	Y (X-ray)	[50, 51]

**Table 2:** Comparison of commonly used biophysical techniques.

Parameter	Biophysical technique		
	X-ray crystallography	NMR spectroscopy	Cryo-EM
MW range of proteins	2–3,000 kDa	60–65 kDa for all-atom; 800–1,000 kDa with sparse labeling	2–3,000 kDa
Time required	Up to several years	Up to 1 year	A few months
Typical resolution range	2–4 Å	N/A	>4 Å
Membrane proteins	Y	Y	Y
Protein dynamics	N	Y	N
Recapitulates physiology	N	Y/N	Y
Artifacts	Crystallization artifacts, single conformation	Reflecting conformational averaging	Possible sample preparation artifacts
Expertise required	Y	Y	Y
Major advantage	Streamlined, high resolution information	Providing information on protein dynamics	Fully functional macromolecular complexes
Major disadvantage	Requiring stable protein crystal that diffracts well	Requiring high concentration sample	Low signal-to-noise ratio for proteins smaller than 300 kDa
